# A DNA Network as an Information Processing System

**DOI:** 10.3390/ijms13045125

**Published:** 2012-04-23

**Authors:** Cristina Costa Santini, Jonathan Bath, Andrew J. Turberfield, Andy M. Tyrrell

**Affiliations:** 1 Department of Electronics, University of York, York YO10 5DD, UK; E-Mail: ccsantini@gmail.com; 2 Clarendon Laboratory, Department of Physics, University of Oxford, Parks Road, Oxford OX1 3PU, UK; E-Mails: j.bath1@physics.ox.ac.uk (J.B.); a.turberfield1@physics.ox.ac.uk (A.J.T.)

**Keywords:** biological networks, information processing, DNA

## Abstract

Biomolecular systems that can process information are sought for computational applications, because of their potential for parallelism and miniaturization and because their biocompatibility also makes them suitable for future biomedical applications. DNA has been used to design machines, motors, finite automata, logic gates, reaction networks and logic programs, amongst many other structures and dynamic behaviours. Here we design and program a synthetic DNA network to implement computational paradigms abstracted from cellular regulatory networks. These show information processing properties that are desirable in artificial, engineered molecular systems, including robustness of the output in relation to different sources of variation. We show the results of numerical simulations of the dynamic behaviour of the network and preliminary experimental analysis of its main components.

## 1. Introduction

Computing is the study of natural and artificial information processes [1]. Natural computing includes the implementation of computational paradigms abstracted from natural phenomena either on traditional electronic hardware or on alternative physical media such as in biomolecular (DNA, RNA) computing [2]. Biocompatible systems that can process information are sought for future biomedical applications. Biomolecular systems are potentially important for these and more general computational applications because of their intrinsically high information storage capabilities and parallelism.

DNA is used to build synthetic molecular machines because the simplicity of its structure and interactions allows control of its assembly through information stored in nucleotide sequences [3,4]. The Watson–Crick double helix is formed by linking together two antiparallel strands of DNA that have complementary base sequences, a process known as hybridization. Attractive interactions between complementary nucleotides contribute to the stability of the structure: adenine (A) on one strand pairs with thymine (T) on the other, while cystosine (C) pairs with guanine (G). It is the remarkable specificity of the interactions between these complementary nucleotides, together with the availability of routine commercial synthesis, that allows DNA to be used as engineering material with which to design complex systems and structures capable of self-assembly and parallel operation [3,4]. This versatile molecule has been used to design machines [5–13], finite automata [14,15], logic gates [16,17], reaction networks [18–20] and logic circuits [21,22], amongst many other structures and dynamic systems. Refer to [3,4,23] for reviews of DNA devices and machines.

Here we present preliminary work towards an autonomous, synthetic DNA network, comprised solely of DNA molecules. This DNA network implements computing paradigms abstracted from natural cellular biochemical reaction networks.

**Figure 1:**
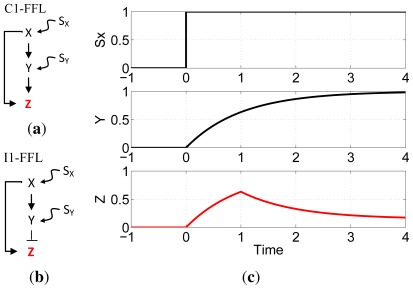
Network motifs. (**a**) Type-1 coherent feed-forward loop (C1-FFL). Arrows denote activation and ⊥ denotes repression. *S_x_* and *S_y_* are input signals that activate transcription of genes X and Y; (**b**) Type-1 incoherent feed-forward loop (I1-FFL); (**c**) Pulse-generation dynamics of the I1-FFL, modelled following reference [33], in response to an ON step of *S_x_* in the presence of *S_y_*. After signal *S_x_* activates gene X, the product of X turns on Z but also its repressor Y. Active Z accumulates until Y levels reach the repression threshold for the Z promoter. As a result, Z production decreases and its concentration drops, resulting in pulse-like dynamics [27,33].

Cellular regulation is achieved through complex networks of interactions among biochemicals and cellular structures. Recurrent network motifs, classifiable in terms of function, architecture, dynamics, or biochemical process [24–26], have been identified. Alon and colleagues investigated transcription networks in the bacterium *E. coli* and the yeast *S. cerevisiae* whose information processing role is to determine the rate of production of specific proteins as a function of the environment [25,26]. In these networks, the nodes are genes and the edges represent transcriptional regulation of one gene by the protein product of another gene. Two important motifs in *E. coli* and *S. cerevisiae* transcription networks are the type-1 coherent feed-forward loop (C1-FFL) and the type-1 incoherent feed-forward loop (I1-FFL). In the C1-FFL (Figure 1a) both paths are positive: X activates both Z and an activator of Z. In the I1-FFL (Figure 1b), the direct path is positive and the indirect path is negative, *i.e.*, X activates Z and an inhibitor of Z. Experimental and computational approaches have shown that the C1-FFL shows sign-sensitive delay that can protect against brief input fluctuations [27], whereas the I1-FFL is responsible for functions such as pulse generation [28], adaptation [29,30], fold-change detection [31] and amplitude filtering [32]. Figure 1c shows an example of pulse generation using the I1-FFL network motif.

Other networks have been investigated and display interesting information processing properties. Acar *et al*. [34] investigate inducibility and network-dosage invariance (*i.e.*, invariance to the number of copies of a gene network in a cell) in the yeast galactose network. Their results revealed that, in general, the presence of two network components, one positive and one negative regulator, is the minimal requirement for network-dosage invariance.

In this work, we develop an implementation of an information processing paradigm abstracted from the yeast galactose network using DNA molecular computation. In particular, we aim to show that a DNA system can be designed and programmed to implement an information processing function that is robust to changes in network dosage and is thus capable of contributing to network behaviour that is reliable despite the stochasticity inherent in molecular systems. We choose to implement, on the DNA network, the basic information processing function of the I1-FFL: pulse generation. We note that the I1-FFL network motif [27] has two components, one positive and one negative regulator, and thus satisfies the minimal requirement for a network whose activity is robust to changes in network dosage [34].

The paper is organized as follows. Section 2 discusses the toolbox, *i.e.*, the DNA processes and structures from which our DNA network is composed. The network itself is described in Section 3. Section 4 contains the results of numerical simulations, and conclusions are presented in Section 5.

## 2. The Toolbox of DNA Processes

**Figure 2:**
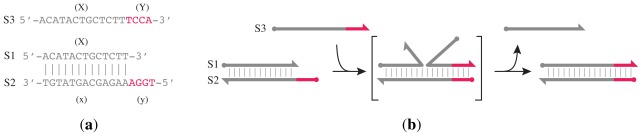
Toehold-mediated strand displacement is a widely used component of DNA reaction networks. (**a**) Nucleotide sequences of three interacting strands, labelled S1, S2, S3, each divided into functional domains that are identified by colour and labelled X, x, Y, y. Pairs of domains (X,x) and (Y,y) are complementary. The double-stranded complex S1•S2 is formed by hybridization of domains X and x of strands S1 and S2 respectively. The short single-stranded domain, y, at the 5′ end of S2 is described as a “toehold”; (**b**) The invading strand, S3, binds to S1•S2 via the toehold to initiate displacement of S1. Strand displacement occurs by means of a random walk of the branch point separating regions of S2 hybridized to S1 and to S3, but it is biased by a decrease in free energy of approximately 2.4 *k_B_T* per base pair [36] when the toehold hybridizes with its complement on the invading strand (a typical toehold is 6 nt in length [35]). Careful design of nucleotide sequences is required to minimize unwanted secondary structure of the component strands in a DNA system. The convention of using a circle and barb to indicate the 5′ and 3′ ends of a strand is used throughout.

Figure 2 illustrates an operation that is often harnessed in the design of dynamic DNA systems: toehold hybridization followed by branch migration resulting in strand displacement [35]. The system depicted in Figure 2 can be considered an information processing system that detects the presence of input S3 and releases output S1 in response. Using the same simple mechanism of toehold-mediated strand displacement, in 2006 Seelig *et al*. [16] showed experimental results for DNA-based digital logic circuits and in 2009, Soloveichik *et al*. [20] discussed reaction cascades with unimolecular and bimolecular kinetics. They illustrate their method, for example, by simulating DNA reaction modules that corresponds to the Lotka–Volterra oscillator.

**Figure 3:**
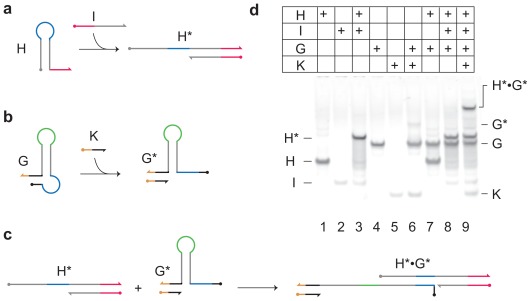
Network components. (**a**) Hairpin loop H has an exposed toehold at the 3′ end (red). Strand I binds to the toehold and invades the neck of the hairpin to activate the loop domain (blue) which can act as a toehold in a downstream strand-displacement reaction; (**b**) The gated hairpin G cannot be opened unless it is first activated by key strand K which acts to reveal the toehold at the base of the neck; (**c**) Once activated, G* can react with the opened hairpin H* to form the complex H*•G*; (**d**) Analysis of reactions of network components using polyacrylamide gel electrophoresis. The open hairpin complex H* is formed when H and I are mixed (lane 3). The open hairpin complex H* does not react with the gated hairpin G (lane 8) unless it has been activated by strand K (lane 9).

Loop-containing DNA structures are interesting elements for information processing systems, as energy and information can be stored in the loop in a form that cannot readily be accessed [37,38]. The neck of the hairpin controls access to the information stored in the loop: the neck is opened using toehold-mediated strand displacement (Figure 3a). Dirks and Pierce [39] demonstrate a system of two hairpins in which the toehold required to open the neck of one is hidden in the loop of the other and *vice versa*. Polymerisation of the loops is triggered when one of the loops is opened by an initiator DNA.

Here, we introduce a new structure, a “gated hairpin loop” (Figure 3), that is used in our DNA network. The gated hairpin (Figure 3b) has a second level of control, *i.e.*, a gate. A second neck controls the accessibility of the toehold used to open the loop and thus to activate the primary loop domain. Preliminary experimental results show that the loop is not opened until the gate is unlocked with the “key” K (Figure 3c). (Please refer to the Supplementary Material for methods and sequences.) The gated hairpin loop is a useful component for the implementation of the DNA network because, before unlocking the “gate”, the information held within this component is not accessible by the other network components.

## 3. The DNA Network Design

**Figure 4:**
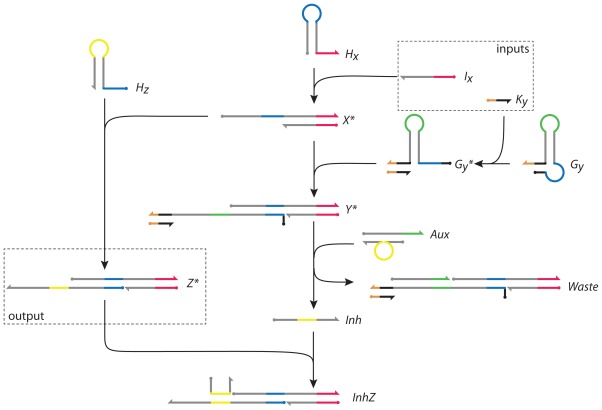
The proposed DNA network. *I_x_* and *K_y_* are inputs to the network; Z^*^ is the output. *H_x_*, *H_z_*, *G_y_* and *Aux* are network components; X^*^, G_y_^*^, Y^*^, *Inh* are intermediate products;*Waste* and *InhZ* are by-products. The steady-state concentration of the output Z^*^ is determined by the branching ratio between the two possible pathways for intermediate X^*^. A pulse in the output of Z^*^ results from a relative delay in the right hand (negative) pathway.

The DNA network is shown in Figure 4. It is abstracted from the topology and functionality of the I1-FFL network motif [27] and network-dosage invariance of the galactose signalling pathway of yeast [34]. Its abstract representation is identical to the I1-FFL shown in Figure 1b. In our DNA implementation, the nodes are complexes of DNA strands. The network has two inputs, *I_x_* and *K_y_*, and its activity is measured by the concentration of the output Z^*^. Its design is based on the principle of toehold-mediated strand displacement (Figure 2) and the gated hairpin structure (Figure 3).

To initiate both positive and negative reaction pathways, input *I_x_* reacts with hairpin *H_x_* (as described in Figure 3a) forming X^*^ and allowing access to the toehold sequestered in the loop of *H_x_* (blue). Following the positive reaction pathway, X^*^ can then react with hairpin *H_z_* to form Z^*^, in which the toehold sequestered in the loop of *H_z_* (yellow) is activated.

Independently, and in parallel, the second input, strand *K_y_*, activates the gated hairpin loop *G_y_* (as described in Figure 3b), forming G_y_^*^. The latter reacts with X^*^ in the negative regulatory pathway, opening the hairpin and forming product Y^*^. Y^*^ binds to double-stranded complex *Aux* in a reaction mediated by its exposed toehold (green), displacing strand *Inh* from *Aux* and forming *Waste*. The displaced single strand *Inh* hybridizes to Z^*^, forming the structure *InhZ* in which the domain that encodes the active state of Z^*^ is inhibited.

The output of the network is measured by the concentration of Z^*^. The toehold activated by production of Z^*^ (the yellow loop domain that is sequestered in hairpin *H_z_*) is reactive and could be used to cascade downstream reactions. It could act as the input signal to another network or could be designed to regulate the production of its own initiator, input strand *I_x_*, providing feedback to the network.

The gated hairpin loop *G_y_* is not active unless input strand *K_y_* is present to open its gate (Figure 3c). In the absence of input *K_y_*, the network only has the positive reaction pathway and input *I_x_* reacts stoichiometrically to produce output Z^*^. In the presence of *K_y_*, through production of G_y_^*^, a proportion of *I_x_* is diverted to the negative reaction pathway, leading to inhibition of Z^*^. The use of the gated hairpin therefore adds an independent control to the output of the network.

Because the two reaction pathways run in parallel, it is important to consider the effects of the timing of the inputs on the network behaviour. We consider two scenarios. In Scenario A, input *K_y_* is added first, such that its reaction with *G_y_* to form G_y_^*^ is substantially complete before the second input *I_x_* is added. In Scenario B, both inputs are added simultaneously. Two effects can contribute to a transient overproduction of output Z^*^ that is later compensated by production of *Inh*, resulting in a pulse of Z^*^. In Scenario B, production of intermediate G_y_^*^ does not begin until both inputs are added, so the initial reaction rate of intermediate X^*^ with G_y_^*^, corresponding to entry into the negative pathway, is zero: this guarantees a pulse of Z^*^. Even in Scenario A, in which both pathways are active when *I_x_* is added, a relative delay in the production of *Inh* resulting from the greater length of the negative pathway gives rise to pulse production.

This network motif is designed to operate far-from-equilibrium, as are the natural biochemical networks that inspired it. The ratios between forward and reverse reaction rates are determined by the free energy changes in each reaction and can be made large by design. Slow reverse reactions will have little effect on transient phenomena (pulse generation). However, if a sufficiently long time is allowed to elapse then reverse reactions—however slow—will ensure that the final state of the system is in thermodynamic equilibrium, independent of the details of reaction pathways. If the output of the network motif cascades forward to actuate downstream processes that are similarly far-from-equilibrium then this equilibrium state is never relevant to its operation.

### 3.1. Programming the DNA Network

In order to achieve a steady-state concentration of output Z^*^ that is invariant to network dosage, *i.e.*, to the concentrations of network components, it is necessary to ensure that the ratio between the time-integrated quantities of input *I_x_* (and therefore of intermediate X^*^) that flow through the positive and negative reaction pathways is invariant. We assume below that the concentrations of network components *H_x_*, *H_z_*, *G_y_* and *Aux* and of input *K_y_* are in sufficient excess over the initial concentration of input *I_x_* that perturbations resulting from the reactions triggered by addition of *I_x_* are small. The rate of activation of the negative pathway depends on the concentration of intermediate G_y_^*^ which is, in general, time-dependent and a function of the input *K_y_*. In Scenario A, when all G_y_^*^ is formed before *I_x_* is added, the time-dependence of the production of *K_y_* plays no part in the behaviour of the network. Network-dosage invariance of the steady-state output is achieved if the concentrations of *H_z_* and the smaller of the concentrations of *G_y_* and of *K_y_* are scaled together ensuring a constant branching ratio between the two reaction pathways. In Scenario B, *K_y_* and *I_x_* are added simultaneously and the time-dependence of the production of G_y_^*^ does affect the output of the network. In this case, network-dosage invariance is achieved if the concentrations of *H_x_*, *H_z_*, *G_y_* and *K_y_* are scaled together (see Supplementary Material). Note that the dynamic component of the network output (the pulse) is not, in general, network-dosage invariant.

The network can be configured in three ways—positive, balanced and negative—defined by its behaviour in Scenario A. The branching ratio between positive and negative pathways is determined by the concentrations of G_y_^*^ and *H_z_* which compete for reaction with intermediate X^*^. In a balanced network, the time-integrated branching ratio is 1:1 and addition of input *I_x_* has no effect on the steady-state concentration of output Z^*^. In an unbalanced network the proportions of *I_x_* that enter the positive and negative pathways are unequal with the result that the output concentration of Z^*^ is changed by addition of *I_x_*: it can be increased or decreased (though not, of course, below zero), depending on the relative concentrations of G_y_^*^ and *H_z_*.

## 4. Simulation Results

We have investigated the behaviour of the proposed DNA network through chemical kinetics simulations. The network was modelled by the following ordinary differential equations:

$$\begin{array}{l} H_{x}+I_{x}\overset{k_{1}}{\to }X^{*}\\ X^{*}+H_{z}\overset{k_{2}}{\to }Z^{*}\\ G_{y}+K_{y}\overset{k_{3}}{\to }G_{y}^{*}\\ X^{*}+G_{y}^{*}\overset{k_{4}}{\to }Y^{*}\\ Y^{*}+Aux\overset{k_{5}}{\to }Inh+Waste\\ Z^{*}+Inh\overset{k_{6}}{\to }InhZ \end{array}$$

All reactions, with the exception of the reaction of *Inh* with Z^*^, involve toehold-mediated strand displacement to open a secondary structure loop. Rate constants *k*_1_ to *k*_6_ are set at 10^5^*M*^−1^*s*^−1^ [35,38]. We have assumed that all reactions are irreversible (rates of reverse reactions can be six orders of magnitude slower for appropriate toehold lengths [40]). Initial concentrations of DNA molecules are specified in Supplementary Material, Table 1: concentrations of network components are of the order of 1 *μ*M. Simulations were performed in Matlab [41] using the ODE solver ode15s.

**Figure 5:**
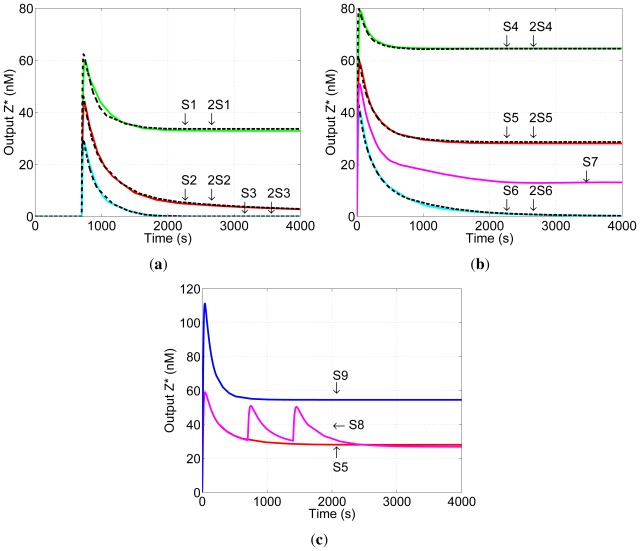
Simulations of the behaviour of the proposed DNA network. Positive (S1,S4), balanced (S2,S5) and negative (S3,S6) networks are shown in green, red and blue respectively. Black dotted lines show results for doubled network dosage. (**a**) Scenario A: input *K_y_* added before *I_x_*. The steady-state concentration of output Z^*^ is non-zero only for the positive networks S1, 2S1; it is independent of network dosage; (**b**) Scenario B: inputs *K_y_*, *I_x_* added simultaneously. In this case the branching ratio is time-dependent, and an initial imbalance between pathways in the “balanced” network results in non-zero steady-state output. Outputs remain robust to network dosage; (**c**) Subsequent addition of input *I_x_*. The steady-state output of the balanced network in Scenario B (S5) increases if the initial input is increased (S9) but is robust to delayed inputs (S8). See text for specifications of network simulations.

Simulation results are shown in Figure 5. The graphs show the concentration of the network output Z^*^ as a function of time. Unless stated otherwise, the initial concentration of input *I_x_* is set to 100 nM. All network components are present at t = 0 s.

Figure 5a shows simulation results for Scenario A (Section 3): input *K_y_* is added first, at t = 0 s, and input *I_x_* is added at t = 700 s when the reaction of *K_y_* with *G_y_* to produce G_y_^*^ is substantially complete. Simulation S1 is unbalanced positively, *i.e.*, the initial concentration of *H_z_* is greater than that of *G_y_* resulting in a non-zero steady-state concentration of output Z^*^. In simulation 2S1, the initial concentrations of all network components are doubled: the concentration of *K_y_* is greater than that of *G_y_* in both cases. The two networks generate similar pulses, and the steady-state outputs of the two networks are the same, demonstrating that the steady-state output of the DNA network is robust to changes in network dosage. The same network behaviour—pulse generation and network-dosage invariant steady-state output—is displayed for balanced (S2 and 2S2) and negative (S3 and 2S3) networks.

Figure 5b shows simulation results for Scenario B: inputs *I_x_* and *K_y_* are delivered simultaneously at t = 0 s. S4-S6 correspond to positive, balanced and negative networks and 2S4–2S6 to the same networks in which the concentrations of network components and input *K_y_* are doubled. Again, under these conditions the steady-state component of the output is robust to the change in network dosage. Note that, as expected, the steady-state output of the balanced network (S5) is different from the zero output of the same network in Scenario A (S2, Figure 5a): this asymmetry is a result of the initial unbalance in the network during production of G_y_^*^. Also shown in Figure 5b is the result of simulation S7 in which all initial concentrations are the same as in 2S5 with the exception of the concentration of *K_y_*, which is as in S5 (half that in 2S5). The outputs of 2S5 and S7 are different, as expected, demonstrating that the concentration of input *K_y_* must be scaled with those of network components in order to achieve network-dosage invariance.

Simulation results shown in Figure 5a,b confirm that the dynamical component of the output Z^*^ (the pulse) changes with network dose. (For an expanded view of the pulses in Figure 5a,b see Figure 1a,b in Supplementary Material.)

The balanced network has another robust behaviour. Once input *K_y_* has had time to react—whether in Scenario A or Scenario B—the steady-state level of output Z^*^ is unchanged by subsequent addition of small quantities of input *I_x_*. Once a steady (and balanced) concentration of G_y_^*^ is established, these subsequent stimuli *I_x_* cause equal activation of the positive and negative reaction pathways, resulting in a pulse of Z^*^ but no change in its steady-state concentration. Figure 5c shows the effects of subsequent additions of input *I_x_*. Simulation S8 is the same as Simulation S5, except that two further quantities of *I_x_* were added after the initial pulse of output Z^*^ had died away. (For a plot of the state space see Figure 2 in Supplementary Material.) The second and third additions of *I_x_* have no effect on the steady-state output of the network, as expected. The results of Simulation S9 are also shown: S9 has the same quantity of input *I_x_* added as in Simulation S8, except that it is all added at once at t = 0 s (initial conditions are otherwise the same as Simulations 5 and 8). The steady-state output in Simulation S9 is different from S5 and S8, demonstrating that the lack of effect of the later additions of *I_x_* is a result of the timing of the inputs and not of saturation of the output of the network.

Figures 3–5 in Supplementary Material present additional simulation results showing robustness of output to subsequent stimuli, the limits of the desired network behaviour and results of extreme imbalance between the activation and repression pathways.

## 5. Conclusions

We have investigated the implementation, in a synthetic DNA reaction network, of computing paradigms abstracted from two different cellular biochemical reaction networks, the yeast galactose network [34] and the type-1 incoherent feed-forward loop network motif [27]. The results of chemical kinetics simulations show that the proposed DNA network can be programmed to implement transient pulse generation with a steady-state output that can be made robust to changes in network dosage. This network has another interesting property: it can be configured such that the steady-state output is proportional to the initial dose of one of the inputs but insensitive to subsequent additions which generate only transient output pulses.

## Supplementary Material
