# Supplementary material for: A DNA Network as an Information Processing System
Source: Int J Mol Sci. 2012 Apr 23;13(4):5125–37. doi: 10.3390/ijms13045125 (PMC3344270; doi:10.3390/ijms13045125)
Supplement: Supplementary file 1 [file ijms-13-05125-s001.pdf]

Int. J. Mol. Sci. **2012**, *13*, 5125-5137; doi:10.3390/ijms13045125

OPEN ACCESS

International Journal of
Molecular Sciences

ISSN 1422-0067

www.mdpi.com/journal/ijms

Article

Supplementary Material—A DNA Network as an Information Processing System

Cristina Costa Santini [1,†], Jonathan Bath [2], Andrew J. Turberfield [2] and Andy M. Tyrrell [1,⋆]

[1] Department of Electronics, University of York, York YO10 5DD, UK; E-Mail: ccsantini@gmail.com

[2] Clarendon Laboratory, Department of Physics, University of Oxford, Parks Road, Oxford OX1 3PU, UK; E-Mails: j.bath1@physics.ox.ac.uk (J.B.); a.turberfield1@physics.ox.ac.uk (A.J.T.)

† Current address: College of Computer and Information Sciences, King Saud University, P.O.Box 22452, Riyadh 11495, KSA.

⋆ Author to whom correspondence should be addressed; E-Mail: andy.tyrrell@york.ac.uk; Tel.: +44-0-1904-322340; Fax: +44-0-1904-322461.

Received: 29 February 2012; in revised form: 31 March 2012 / Accepted: 17 April 2012 / Published: 23 April 2012

Abstract: Biomolecular systems that can process information are sought for computational applications, because of their potential for parallelism and miniaturization and because their biocompatibility also makes them suitable for future biomedical applications. DNA has been used to design machines, motors, finite automata, logic gates, reaction networks and logic programs, amongst many other structures and dynamic behaviours. Here we design and program a synthetic DNA network to implement computational paradigms abstracted from cellular regulatory networks. These show information processing properties that are desirable in artificial, engineered molecular systems, including robustness of the output in relation to different sources of variation. We show the results of numerical simulations of the dynamic behaviour of the network and preliminary experimental analysis of its main components.

Keywords: biological networks; information processing; DNA

---

1. The Gated Hairpin Loop

*Sequences**

H: ACACAACACACACCACACAGATCCGTGTGGTGTGTGTTGTGTCCGCTC
I: GAGCGGACACAACACACACCACAC
G: GCGCTCGGATCTGTGTGGTGTGTGTTGTGTCCTTCAACACAACACACACCACACGAGCGCTTTAGA
K: CCCCCCTCTAAAGCGCTCG

* note that strand K has an extra domain, CCCCCC, at the 5′ end which can be used as a toehold to facilitate removal of K by addition of its complement. This feature was not used for the experiments presented here, nor is it necessary for construction of the network.

Method

All strands were purchased from IDT and used without further purification. Stocks of each component strand (1.0 μM *H*, 2.0 μM *I*, 0.8 μM *G* and 1.6 μM *K*) were quenched by heating to 96 °C for 5 min then transfering to ice. Reactions (20 μL) containing 2 μL of appropriate stock solutions in 10 mM Tris-HCl, 50 mM NaCl and 10 mM MgCl2 (pH 8.0) were incubated for 1 h at room temperature. Samples were run on a 15 % polyacrylamide gel (29:1) in TAE (40 mM Tris, 20 mM acetic acid, 1 mM EDTA) at 222 V, 4 °C for 1 h, then stained with sybr gold.

2. Network Dose Invariance, Scenario B

Definitions

$G_{y0} \equiv G_y(0)$ is the initial concentration of network component G_y;
G_{yr0} is a (constant) reference initial concentration for this component.
Initial and reference concentrations for network components H_x, H_z and for input K_y, and an initial concentration for input I_x, are defined similarly.

Reaction Equations

The steady-state output of the network is governed by the branching ratio between the positive and negative reaction pathways. The steady-state output is equal to the difference between the time-integrated quantities of intermediate X^* that are converted initially to output Z^* and to intermediate Y^*, as Y^* will eventually contribute stoichiometrically to the inhibition of Z^*. (The time-dependence of the conversion of Y^* to *Inh* and the sequestration of Z^* by *Inh* affect the pulse shape but not the steady-state output.) These quantities are governed by the following coupled rate equations:

$$\frac{d}{dt}H_x(t) = -k_1 I_x(t) H_x(t) \tag{S1}$$

$$\frac{d}{dt}H_z(t) = -k_2 X^*(t) H_z(t) \tag{S2}$$

$$\frac{d}{dt}G_y^*(t) = k_3 G_y(t) K_y(t) - k_4 X^*(t) G_y^*(t) \tag{S3}$$

$$\frac{d}{dt}G_y(t) = \frac{d}{dt}K_y(t) = -k_3G_y(t)K_y(t) \tag{S4}$$

$$\frac{d}{dt}I_x(t) = -k_1I_x(t)H_x(t) \tag{S5}$$

$$\frac{d}{dt}X^*(t) = k_1I_x(t)H_x(t) - k_2X^*(t)H_z(t) - k_4X^*(t)G_y^*(t) \tag{S6}$$

$$\frac{d}{dt}Y^*(t) = k_4X^*(t)G_y^*(t) \tag{S7}$$

$$\frac{d}{dt}Z^*(t) = k_2X^*(t)H_z(t) \tag{S8}$$

In Scenario B the boundary conditions are:

$$G_y^*(0) = 0; G_y(0) = G_{y0}; K_y(0) = K_{y0}; H_x(0) = H_{x0}; H_z(0) = H_{z0};$$
$$I_x(0) = I_{x0}; X^*(0) = 0; Y^*(0) = 0; Z^*(0) = 0.$$

We assume that the time-dependent concentrations of network components (including G_y, H_x and H_z) and of input K_y are not sigificantly perturbed by addition of input I_x. In this limit Equations (S1) to (S3) become:

$$\frac{d}{dt}H_x(t) = 0 \tag{S9}$$

$$\frac{d}{dt}H_z(t) = 0 \tag{S10}$$

$$\frac{d}{dt}G_y^*(t) = k_3G_y(t)K_y(t) \tag{S11}$$

We further assume that the initial concentrations of network components and of input K_y are scaled by a constant α relative to a reference network. For network components and K_y we introduced scaled functions

$$G_y^*(t) = \alpha G_{yr}^*(t'); G_y(t) = \alpha G_{yr}(t'); K_y(t) = \alpha K_{yr}(t');\ H_x(t) = \alpha H_{xr}(t'); H_z(t) = \alpha H_{zr}(t') \tag{S12}$$

where $t' = \alpha t$. Trivially, $G_{y0} = \alpha G_{yr0}$, $K_{y0} = \alpha K_{yr0}$ and, in the limit of small I_x, $H_{xr}(t') = H_{xr0}$, $H_{zr}(t') = H_{zr0}$. For input I_x (which is not scaled) and for intermediates X^*, Y^* and Z^* we write:

$$I_x(t) = I_{xr}(t'); X^*(t) = X_r^*(t'); Y^*(t) = Y_r^*(t'); Z^*(t) = Z_r^*(t') \tag{S13}$$

Equations (S4) to (S11) become:

$$\frac{d}{dt'}G_{yr}(t') = \frac{d}{dt'}K_{yr}(t') = -k_3G_{yr}(t')K_{yr}(t') \tag{S14}$$

$$\frac{d}{dt'}I_{xr}(t') = -k_1I_{xr}(t')H_{xr}(t') \tag{S15}$$

$$\frac{d}{dt'}X_r^*(t') = k_1I_{xr}(t')H_{xr}(t') - k_2X_r^*(t')H_{zr}(t') - k_4X_r^*(t')G_{yr}^*(t') \tag{S16}$$

$$\frac{d}{dt'}Y_r^*(t') = k_4X_r^*(t')G_{yr}^*(t') \tag{S17}$$

$$\frac{d}{dt'}Z_r^*(t') = k_2X_r^*(t')H_{zr}(t') \tag{S18}$$

$$\frac{d}{dt'}H_{xr}(t') = 0 \tag{S19}$$

$$\frac{d}{dt'}H_{zr}(t') = 0 \tag{S20}$$

$$\frac{d}{dt'}G^*_{yr}(t') = k_3 G_{yr}(t')K_{yr}(t') \tag{S21}$$

In the case $\alpha = 1$ (*i.e.*, $t' = t$) these are identical to the equations describing the evolution of the reference network, and the boundary conditions are also identical. The dependence of the scaled functions defined by Equations (S12) and (S13) on the scaled time variable t' is therefore the same as for the reference network for all network scalings. We note that $Y^*(\infty) = Y^*_r(\infty)$ and $Z^*(\infty) = Z^*_r(\infty)$, independent of α. The time-integrated quantities of intermediate X^* that are converted initially to Y^* and to Z^* ($Y^*(\infty)$, $Z^*(\infty)$ respectively) are therefore independent of network scaling.

Conclusion

If the initial concentrations of network components G_y, H_x, H_z and *Aux* and of input K_y are sufficiently large that the network is not significantly perturbed by addition of the input I_x AND if inputs I_x AND K_y are added simultaneously (Scenario B) AND if initial concentrations G_{y0},H_{x0}, H_{z0} and K_{y0} are kept in the same ratio THEN the branching ratio of I_x into the positive and negative pathways and the steady-state network output are independent of network dosage.

Figure S1. The same plots shown in Figures 5a and 5b on an expanded time scale showing the dynamical component of the output, the pulse. It is possible to see at this scale that the pulse rise time, fall time, width and amplitude do change in response to changes in network dosage.

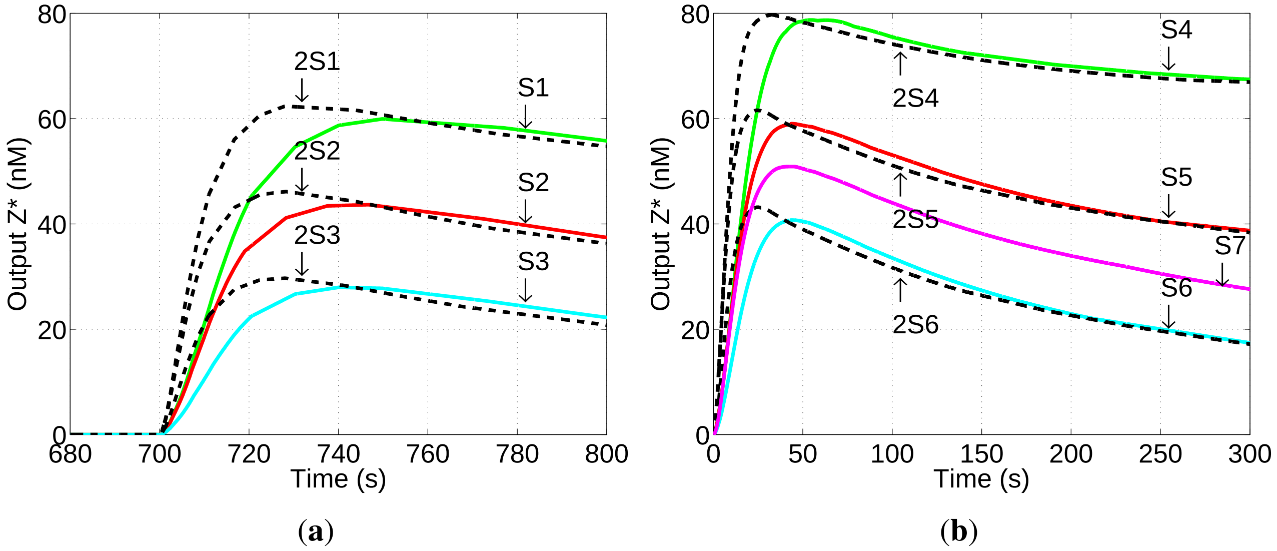

Figure S2. Comparison between dynamical and steady-state components of the output for simulations S5, 2S5 (two networks with different component dosages) and S8 (multiple stimuli) (Figure 5). (**a**) Output Z^* as a function of time; (**b**) Phase plane with coordinates corresponding to concentrations of components Z^* and Inh. Key: S5 (red), 2S5 (dotted black); (**c**) Phase plane with coordinates corresponding to concentrations of components Z^* and Inh. Key: S8 (pink). In the phase plane it is easier to visualize the variation in the dynamical component of the output as well as the invariance to this dosage variation in the steady-state (the trajectories converge at [*Inh*] = 0).

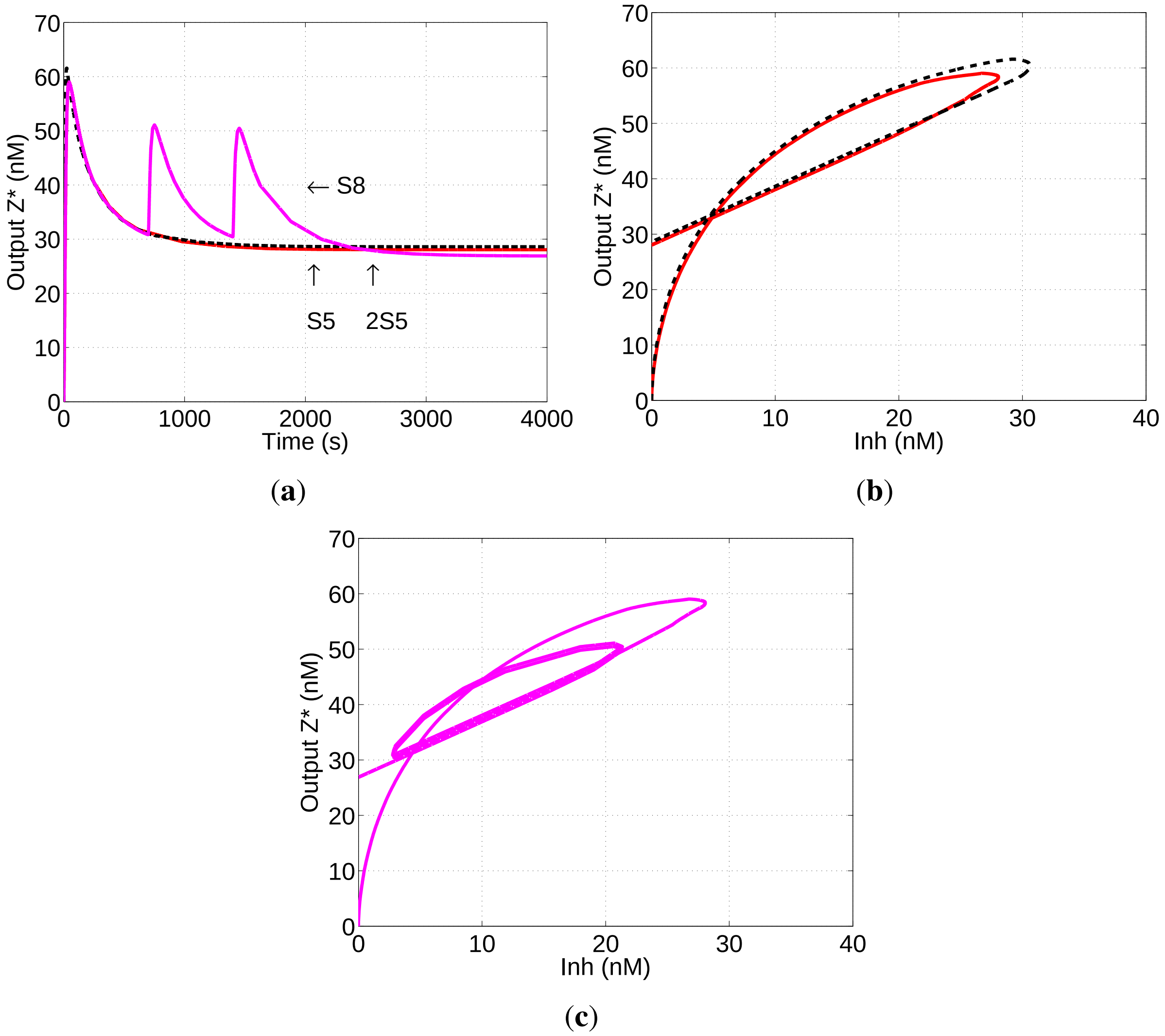

Figure S3. Simulation results showing proportionality between output and input, and robustness of output to subsequent stimuli. (**a–d**) Simulations S5, S8 and S9 (as Figure 5c). Key: S5 (red), S8 (pink), S9 (blue). (**a**) Output Z^*, as Figure 5c; (**b**) Z^* (solid lines), I_x (dotted lines); (**c**) Z^* (solid lines), G_y^* (dotted lines); (**d**) H_z (solid lines), G_y^* (dotted lines); (**e–f**) as (**a–d**) but with Simulation S10 substituted for S8. Key: S5 (red), S10 (pink), S9 (blue). Simulation S10 is similar to S8 but with larger subsequent stimuli: the initial input stimulus of 100 nM I_x is followed by stimuli that raise the concentration of I_x by 200 nM at 700 s and by 200 nM at 1400 s. Even with this input dose, the concentration of network components is sufficient to ensure that the steady state activation of the network is still very close to that achieved with the same initial stimulus in S5 and S8. Note that in both S8 and S10, the additional inputs of I_x at t = 700 s and t = 1400 s are followed by a drop in H_z that exactly matches the drop in G_y^*. For this reason the subsequent stimuli causes no increase in the steady-state level of activation of the network.

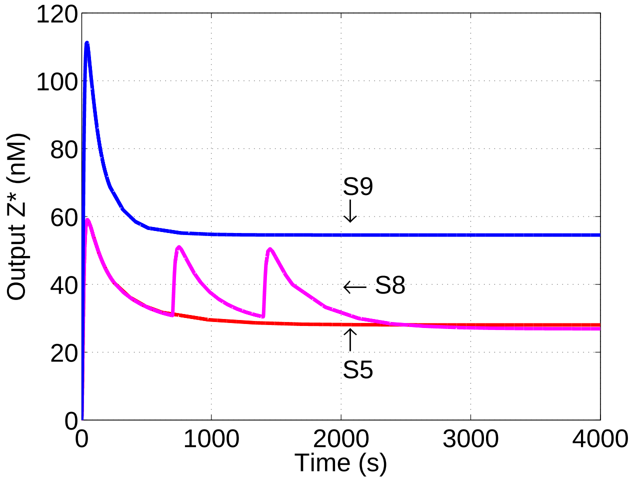

(**a**)

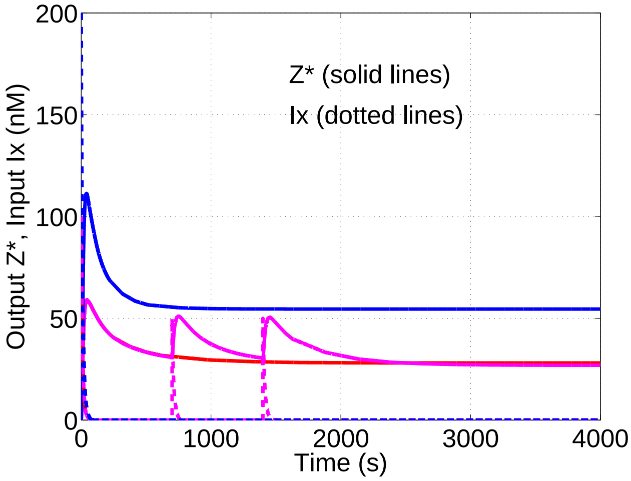

(**b**)

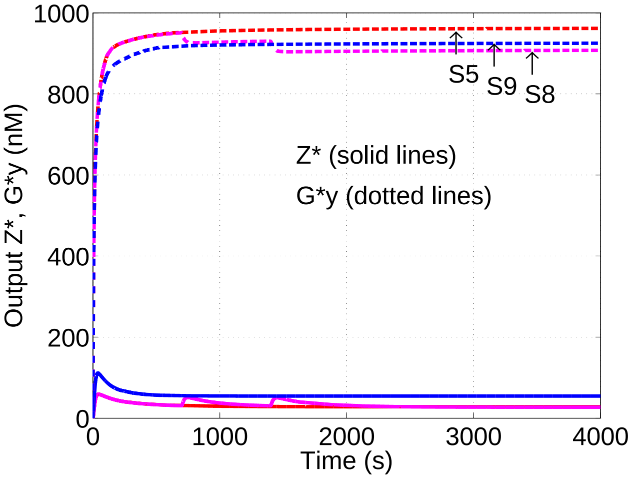

(**c**)

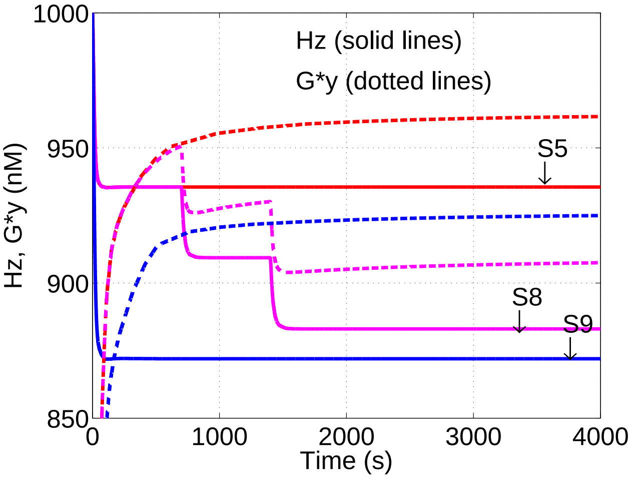

(**d**)

Figure S3. *Cont.*

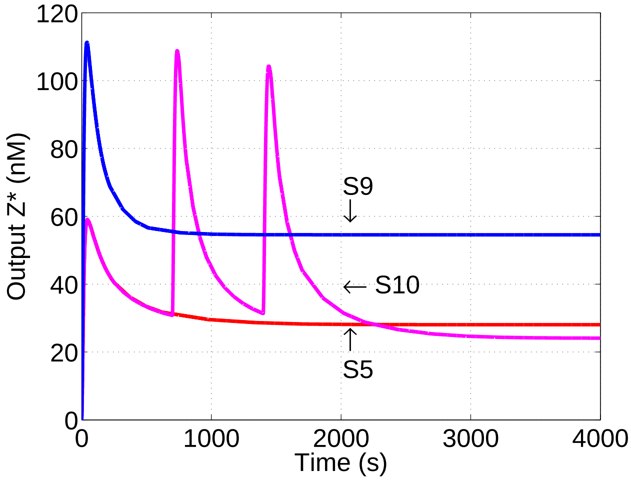

(e)

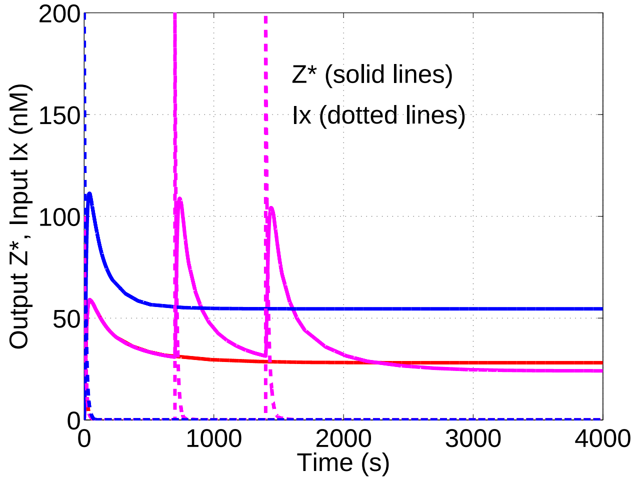

(f)

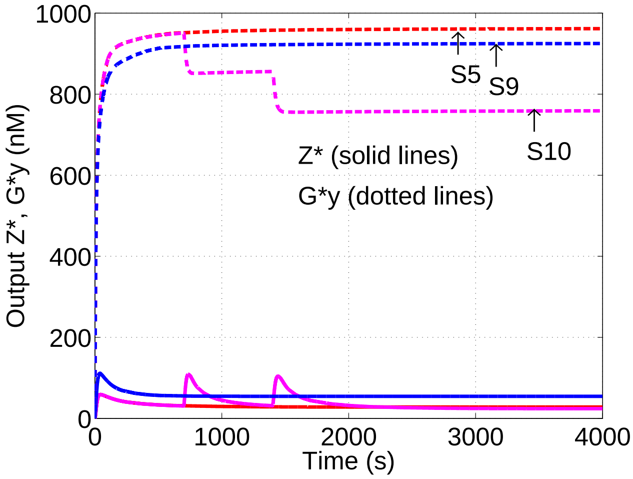

(g)

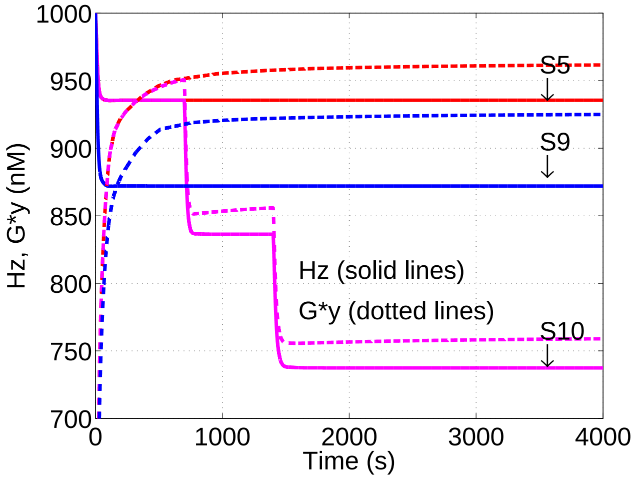

(h)

Figure S4. Simulations S11, S12 and S13, showing the limits of the desired network behaviour. (**a**) In Simulations S11, S12 and S13 the concentrations of network components and K_y were reduced in concentration relative to those in S5 by factors of 2, 5 and 10 respectively. The initial concentration of input I_x was maintained at 100 nM. The steady-state level of Z^* in S12 (2-fold excess of network over input) remains very close to the steady-state level of Z^* in S5. However, in S13 (no excess network concentration) there is a significant change in output level, supporting the conclusion that in order to maintain network-dosage invariance, the network components have to be in significant excess in relation to the input stimulus I_x; (**b**) Simulation S14 is as S11, except for additional stimuli that increased the concentration of input I_x by 250 nM at t = 700 s and 250 nM at t = 1400 s. The total input dose (equivalent to 600 nM) is greater than the initial network concentration. The steady-state output deviates from that of S11, demonstrating that robustness to subsequent input stimuli is only observed if network components remain in excess; (**c**) As (**b**), with in addition the concentration of I_x (dotted lines).

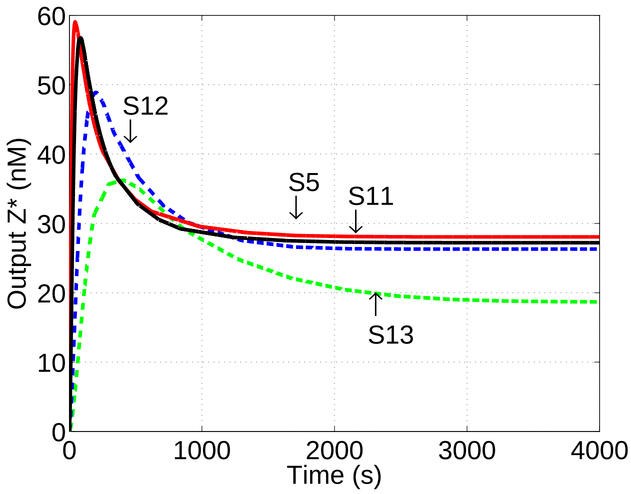

(**a**)

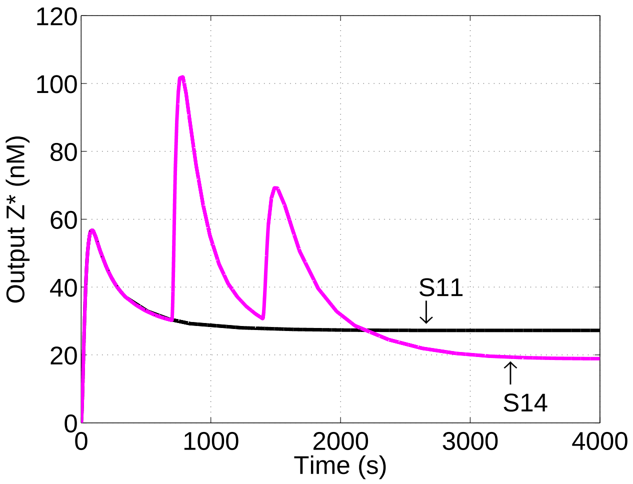

(**b**)

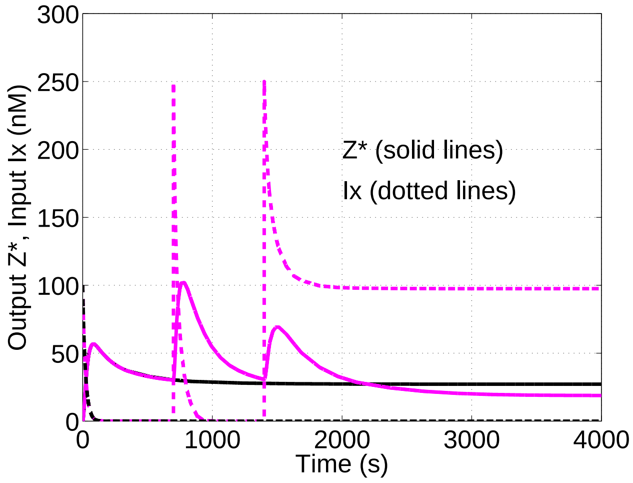

(**c**)

Figure S5. Simulation results showing the results of extreme unbalance between the activation and repression pathways. (**a**) Simulation S2 (as Figure 5a). At t = 0 s all components of the network (H_x, H_z, G_y and Aux) as well of input K_y were combined with initial concentrations of 1 μM. Input stimulus I_x (100 nM) was added at t = 700 s. In this case, the reaction of K_y with G_y to produce repressor complex G_y^* is complete, balancing the repression and activations pathways, before input of I_x. The network therefore shows no steady-state activation. The transient output pulse is explained by the relative delay of the inhibition pathway, which has a longer chain of interactions; (**b**) Simulation S15 is the same as S2 except that input K_y is zero, so the negative reaction pathway is not activated. All input stimulus I_x reacts with H_z leading to a stoichiometric yield of output Z^*.

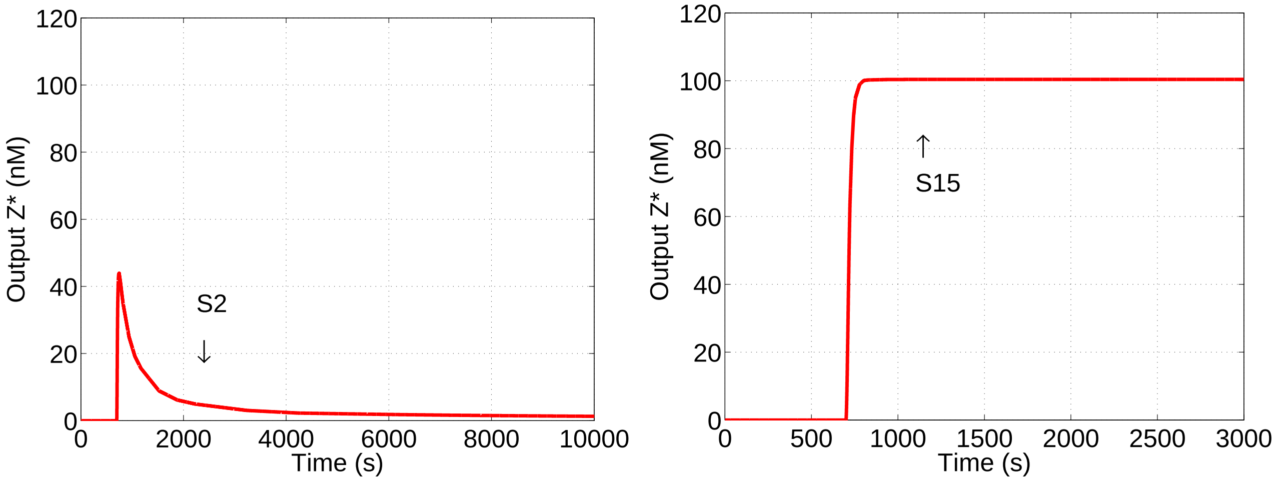

(**a**) (**b**)

Table S1. Initial concentrations and output Z^* at t = 4000 s for all the simulations discussed. For most simulations this is the steady-state level of the output. For simulation S2, for example, the output Z^* slowly tends to zero, as shown in Supplementary Figure 5.

**Simulation**	**t = 0 s**						**t = 700 s**	**t = 1,400 s**	**t = 4,000 s**
	Hz **(nM)**	***Hx*** **(nM)**	***Gy*** **(nM)**	***Aux*** **(nM)**	***Ky*** **(nM)**	***Ix*** **(nM)**	***Ix*** **(nM)**	***Ix*** **(nM)**	***Z**** **(nM)**
**S1**	1,000	1,000	500	1,000	3,000	0	100	0	32
**2S1**	2,000	2,000	1,000	2,000	3,000	0	100	0	33
**S2**	1,000	1,000	1,000	1,000	3,000	0	100	0	2.7
**2S2**	2,000	2,000	2,000	2,000	3,000	0	100	0	2.8
**S3**	500	1,000	1,000	1,000	3,000	0	100	0	0
**2S3**	1,000	2,000	2,000	2,000	3,000	0	100	0	0
**S4**	1,000	1,000	500	1,000	500	100	0	0	64
**2S4**	2,000	2,000	1,000	2,000	1,000	100	0	0	64
**S5**	1,000	1,000	1,000	1,000	1,000	100	0	0	28
**2S5**	2,000	2,000	2,000	2,000	2,000	100	0	0	28
**S6**	500	1,000	1,000	1,000	1,000	100	0	0	0.1
**2S6**	1,000	2,000	2,000	2,000	2,000	100	0	0	0.2

Table S1. *Cont.*

Simulation	t = 0 s						t = 700 s	t = 1,400 s	t = 4,000 s
	Hz (nM)	*Hx* (nM)	*Gy* (nM)	*Aux* (nM)	*Ky* (nM)	*Ix* (nM)	*Ix* (nM)	*Ix* (nM)	*Z** (nM)
**S7**	1,000	1,000	1,000	1,000	2,000	100	0	0	13
**S8**	1,000	1,000	1,000	1,000	1,000	100	50	50	26
**S9**	1,000	1,000	1,000	1,000	1,000	200	0	0	54
**S10**	1,000	1,000	1,000	1,000	1,000	100	200	200	24
**S11**	500	500	500	500	500	100	0	0	27
**S12**	200	200	200	200	200	100	0	0	26
**S13**	100	100	100	100	100	100	0	0	18
**S14**	500	500	500	500	500	100	250	250	18
**S15**	1,000	1,000	1,000	1,000	0	0	100	0	100

© 2012 by the authors; licensee MDPI, Basel, Switzerland. This article is an open access article distributed under the terms and conditions of the Creative Commons Attribution license (http://creativecommons.org/licenses/by/3.0/).
